# New Zealand medical students’ views of euthanasia/assisted dying across different year levels

**DOI:** 10.1186/s12909-021-02558-2

**Published:** 2021-02-23

**Authors:** Luke Nie, Kelby Smith-Han, Ella Iosua, Simon Walker

**Affiliations:** University of Otago, Dunedin, New Zealand

**Keywords:** Assisted dying, Euthanasia, Medical students, Medical education, Mixed method, Quantitative, Qualitative, Policy design

## Abstract

**Background:**

Previous studies report a majority of the general public support euthanasia/assisted dying (EAD), while a majority of doctors are opposed. In considering policy decisions about EAD, some may discount the views of doctors because they take them to be based on personal values or tradition, rather than reasons that the general public might share. One way to explore this notion is to examine whether medical students’ views change during their medical education.

The objective of this study was to learn how New Zealand medical students view EAD and whether students at different year levels have different views.

**Methods:**

An on-line survey of undergraduate medical students was conducted asking whether they supported a law change to allow EAD. Quantitative data was analysed using unadjusted and multiple logistic regression. Thematic analysis was conducted with the qualitative data.

**Results:**

A total of 326 students replied to the survey. The overall response rate was 28%. 65% of 2nd year students were supportive of EAD, compared to 39% in 5th year. The odds of 5th year students supporting a law change compared to 2nd year was 0.30 (95% CI: 0.15–0.60).

The predominant themes found in the qualitative results indicate that medical students support or oppose EAD for reasons similar to those found in the wider debate, and that their views are influenced by a range of factors. However, several at all year levels cited an aspect of medical school as having influenced their views. This was mentioned by participants who were supportive of, opposed to, or unsure about EAD, but it was the type of influence most often mentioned by those who were opposed.

**Conclusions:**

The quantitative findings show students at the end of 5th year were less likely to support EAD than students at the end of 2nd year. We suggest that this difference is most likely due to their time in medical education. This suggests that the lower support found among doctors is in part related to medical education and medical work rather than age, personality, or social context. The qualitative findings indicate that this is not related to a particular educational experience at Otago Medical School but a range.

**Supplementary Information:**

The online version contains supplementary material available at 10.1186/s12909-021-02558-2.

## Introduction

Euthanasia or assisted dying (EAD)^1^ is a long-standing and highly contentious issue in medical ethics. Internationally, numerous studies have reported majority support for EAD among the general public but lower and often minority support among doctors [1, 2]. A recent study synthesizing the evidence on public opinion in New Zealand calculated a weighted average of 68% support for EAD [3]. In comparison, a study of New Zealand general practitioners found 41% [3]. One study of Australian doctors reported that 75% were opposed to EAD, while 87% would be unwilling to personally participate in the practice if it became legal [4]. Other studies show that the doctors most opposed are palliative care specialists [5–7].

In recent years a number of countries and states have legalized various forms of EAD [1]. Currently, EAD is illegal in New Zealand, but in September of 2020 a binding referendum was held on an Act that would make it legal. This Act received majority support and will come into force in November 2021. In a democracy such as New Zealand this may seem the most appropriate way to resolve longstanding and contentious policy disputes. Furthermore, the principle of autonomy, which has become very influential in medical ethics internationally (including New Zealand) [8], may suggest that the views of patients, i.e. the general public, should have a role in determining the kinds of health services that are available. At the same time, it is important that policy decisions are informed by people with expert knowledge of the issue – in this case doctors – as they may be aware of implications that a policy may have which non-experts do not see. Moreover, it is possible that if the general public knew what doctors know, they may also be less likely to support EAD. Then again, the views of experts may be discounted if they are thought to be based on reasons that are not shared by the general public. It is possible, for example, that doctors are more likely to be opposed to EAD because they are more religious [9, 10], or less ethnically representative of society [10].

One way to examine whether doctors’ views of EAD are based on their individual values or reasons that could be shared amongst the general public is to examine the views of medical students at different stages of learning. Medical students are in a transitional state between the general public and doctors. Exploring their views, and whether they change as they progress through medical training, may illuminate how doctors’ views on EAD are formed. In addition, it may indicate how the future workforce of doctors will be affected by EAD legislation, for example, by indicating whether more or less doctors will consider the practice ethically troubling, and how many may be willing to participate in the process.

Internationally, studies of medical students’ views on EAD report a variety of results. These studies are highly variable in design and thus are difficult to compare directly. Several indicate that medical students are more supportive of EAD than doctors [11, 12], and multiple studies find majority support among medical students (South Africa, Munich, Switzerland) [7, 13, 14]. In contrast, two studies from Poland and the UK found a majority were opposed [15, 16]. One study surveying medical students at an Austrian university at three time points suggests that the finding of increased support over time reflects the increase in support in Austrian society [17]. Societal factors may contribute to the variability in the levels of support found across countries. For example, the lower level of support in Poland is possibly a reflection of the importance of Catholicism in Poland, which is traditionally opposed to EAD. Religious belief has been consistently associated with lower support for EAD [1]. In comparison, the higher level of support in Switzerland may reflect a greater cultural acceptance, connected to the fact that assisted suicide has been legal and practiced there for many years. A comparative study of Belgian students reported that philosophy students and law students are more likely than medical students to ‘unconditionally accept euthanasia’ with 56 and 47% respectively, compared to 31% for the medical students [18].

A small number of studies in other countries have investigated how medical education affects student views. A 1995 study at the Puerto Rico School of Medicine found that 83% of 1st year students were supportive of “alleviat [ing] suffering when they cannot treat successfully an illness and death is imminent” compared to 61% of 3rd year students [19]. A survey undertaken by Clemens et al., distributed among German medical students, found that 45% of 3rd year students supported EAD, compared to approximately 35% of 6th year students [20]. A similar difference was reported in a study of medical students at the Chinese University of Hong Kong, which found that 50% of 1st year students supported EAD, compared to 23% of 5th year students [21]. In contrast, a survey of first and final year medical students at an English Medical School, designed to investigate how religion affects students’ views of end-of-life decisions, found that being a final year student was weakly associated with a greater likelihood of agreeing with EAD [16]. However, this study found that the majority of students were opposed to EAD at both stages of learning. Overall, these studies appear to show that medical education makes people less likely to support EAD.

In order to understand why doctors are more likely to oppose EAD than the general public, this study undertook to learn how New Zealand medical students view EAD and whether their views are different at different stages of their medical education.

## Methods

The study used an online survey with quantitative and qualitative elements to learn whether medical students at Otago Medical School support, oppose, or are unsure about EAD being made lawful, the reasons they hold those views, and experiences that they identified as having influenced them.

### Participants

The Otago Medical School program is 6 years long and includes a competitive Health Sciences First Year (HSFY), 2 years of Early Learning in Medicine (ELM), and three clinical years of Advanced Learning in Medicine (ALM). Around 70% of students enter the program from HSFY, usually following secondary education, while the remaining 30% of students enter ELM after completion of an undergraduate degree or through an ‘Alternative’ category, comprising older applicants from a range of backgrounds, such as other health related professions [22].

All medical students at Otago Medical School during the end of the 2018 academic year were invited to participate in the research. The listed year categories are for those who have completed that specified year, due to the timing of the research. Those who graduated at the end of 2018 were excluded because they ceased being students while the study was undertaken and so could not be contacted through the University system. A Qualtrics questionnaire was distributed via student email addresses and official class Facebook groups. A prize draw incentive of two $50 supermarket vouchers was offered. Participation was voluntary and anonymous, though to participate in the draw respondents were asked to provide their student ID number. Two subsequent reminders were sent, spaced 2–3 weeks apart.

### Questionnaire

The questionnaire was constructed to learn whether the students support, oppose or are unsure about EAD, what informed these views, and the reasons behind them (see appendix). Demographic information about participants’ ethnicity, religion, age, gender and prior training was collected as part of the survey.

The first question in the survey (Q1) was as follows:

‘Do you think the law in New Zealand should be changed to allow doctors, under certain circumstances, to provide or administer a medicine to a person, at their voluntary and competent request, that will bring about their death?’

To minimise the problem of framing bias, this question was taken from a recent ‘Citizens’ Jury’ project concerning New Zealanders’ views on EAD [23]. As part of the design of this Citizens' Jury, the wording of this question had been discussed and approved by a steering group of four recognised experts with a mix of views on whether EAD should be legal [23].

### Analysis

The chi-squared goodness-of-fit test was used to assess the representativeness of the survey respondents to the medical school cohort within each year. Monte Carlo exact tests were alternatively used for expected counts < 5. Associations between views on EAD and year of medical education were investigated using both unadjusted and multiple logistic regression. The multiple regression model adjusted for potential confounders including gender, Māori ethnicity, and religiosity. Binary views on EAD were measured using two different outcomes: Yes versus Unsure/No and No versus Unsure/Yes. Variance inflation factors were investigated for the presence of collinearity. Stata software version 16.0 was used for all statistical analyses. The two-sided significance level α = 0.05 was specified for all statistical tests.

Responses to questions about the reasons behind participants’ responses to question one, and what particular experiences had influenced their thinking on this issue (question 2, section 2 in the survey), were analysed using the general inductive approach [24]. Independent parallel coding and creation of initial themes was developed by LN, with SW as the second coder. Next, axial coding was used relating concepts to each other and collating the codes into substantial themes by LN and SW. Continuous revision of the themes were refined by LN, SW and KSH. Microsoft Excel 2016 was used for the collating and revision. Counter-arguments about the delegation of themes led to continued discussion with LN, SW, and KSH until concordance was achieved. The themed responses were then grouped into years 2 and 3 (ELM) and years 4 and 5 (ALM), to identify whether students with clinical experience offered different responses.

## Results

### Quantitative results

A total of 326 of the 1152 students contacted responded to the survey, giving a total response rate of 28% (326/1152). The response rates for the earlier years are higher than the latter years (2nd 35%, 105/296; 3rd 31%, 91/290; 4th 22%, 66/296; 5th 24%, 64/270). Independent demographics (Appendix, Table 6) are similar across the years with respect to ethnicity and religiosity. Gender distribution is similar across 2nd, 3rd, and 4th years but in the 5th year there is a relative increase in the number of male respondents. Obviously, as there is a progression through the years of medical school education, there are more respondents in the 25+ age group category. The goodness-of-fit tests showed that within each of the 4 years, the survey respondents were representative with respect to age, ethnicity (Māori/Non-Māori), and gender (Male/Female) (Appendix, Table 7). Information about the religious affiliation of students is not kept by the Medical School, but comparison can be drawn with the general population. 68% of participants reported having ‘no religion’ compared to 52% of the general population and 58% of those aged 15–29 in the general population, as shown in the 2018 national census data [25]. Ninety-three percent (304/326) of the participants were aged 18–29 (see Appendix, Table 6). This indicates that the participants were less religious as a group than the general population, including those in the same age bracket. However, it is unknown whether they were more or less religious than the rest of the cohort. If there is any sampling bias, it would be reasonable to expect this to apply across year levels, and so the comparison between those year levels would still hold.

The responses to Q1 (section 2) are provided in Table 1. Table 1 shows an overall 56% (184/326) support for the legalization of EAD, while 22% (71/326) are unsure, and 22% (71/326) oppose. The amount of support is lower at 5th year compared to the earlier years, at 65% among those at 2nd year, 63% at 3rd year, 52% at 4th year, and 39% at 5th year. Accordingly, opposition rates are higher at 5th year than other years, at 14, 20, 24 and 34% respectively.

**Table 1:** Participant year levels and answers to question 1 (section 2)

Q1: *‘Do you think the law in New Zealand should be changed to allow doctors, under certain circumstances, to provide or administer a medicine to a person, at their voluntary and competent request, that will bring about their death?’*					
Year levels	Yes	No	Unsure	Total	Response rate
2nd	68 (64.8%)	15 (14.3%)	22 (21.0%)	105	35%
3rd	57 (62.6%)	18 (19.8%)	16 (17.6%)	91	31%
4th	34 (51.5%)	16 (24.2%)	16 (24.2%)	66	22%
5th	25 (39.1%)	22 (34.4%)	17 (26.6%)	64	23%

For the support analyses (Table 2), the univariate and adjusted models produced similar estimates and inferences: both religiosity and year of study were associated with a decreased odds of supporting EAD. Specifically, after adjustment for the potential confounders, medical students in their 5th year of training had 0.30 (CI: 0.15, 0.60; *p* = 0.001) and 0.33 (CI: 0.16, 0.68; *p* = 0.003) times the odds of supporting compared to students in their 2nd and 3rd years respectively.

**Table 2:** Unadjusted and Adjusted Odds Ratios (OR) for Potential Predictors of Support Euthanasia/Assisted Dying, with 95% Confidence Intervals (CI) and *p*-values

Demographic *(Reference category)*	*Unadjusted*			*Adjusted*		
	*OR*	*95% CI*	*p-value*	*OR*	*95% CI*	*p-value* ^*a*^
Year of Study [2]			**0.006** ^***a***^			**0.004** ^***a***^
3	0.91	0.51, 1.64	0.758	0.91	0.48, 1.71	0.767
4	0.58	0.31, 1.08	0.087	0.57	0.29, 1.13	0.106
5	0.35	0.18, 0.66	**0.001**	0.30	0.15, 0.60	**0.001**
Gender *(Female)*						
Male	1.02	0.82, 1.28	0.842	1.04	0.62, 1.72	0.893
Ethnicity *(Non-Māori)*						0.543
Māori	1.16	0.63, 2.11	0.638	0.98	0.51, 1.90	0.953
Religiosity *(No)*						
Yes	0.22	0.13, 0.36	**< 0.001**	0.22	0.13, 0.37	**< 0.001**

^*a*^ For overall group differences

The opposition results (Table 3) were also predominantly comparative between the unadjusted and adjusted logistic regression models with religiosity and year of study again being presented as significant variables. After adjustment, medical students in their 5th year of training were associated with a 260% (OR = 3.60; CI: 1.53, 8.50; *p* = 0.003) increase in the odds of opposing EAD compared to 2nd year medical students. Although the univariate model suggested that 5th Year medical students were also more likely to oppose than their 3rd year counterparts (OR = 2.12; CI: 1.02, 4.41; *p* = 0.043), this significant association no longer remained after accounting for all covariates (OR = 2.08; CI: 0.90, 4.83; *p* = 0.087).

**Table 3:** Unadjusted and Adjusted Odds Ratios (OR) for Potential Predictors of Opposition to Euthanasia/Assisted Dying, with 95% Confidence Intervals (CI) and *p*-values

Demographic *(Reference category)*	*Unadjusted*			*Adjusted*		
	*OR*	*95% CI*	*p-value* ^*a*^	*OR*	*95% CI*	*p-value* ^*a*^
Year of Study [2]			**0.024** ^***a***^			**0.032** ^***a***^
3	1.48	0.70, 3.14	0.307	1.73	0.75, 3.97	0.198
4	1.92	0.88, 4.21	0.103	2.15	0.90, 5.15	0.085
5	3.14	1.48, 6.66	**0.003**	3.60	1.53, 8.50	**0.003**
Gender *(Female)*						
Male	1.11	0.85, 1.46	0.426	1.45	0.77, 2.71	0.251
Ethnicity *(Non-Māori)*						
Māori	0.52	0.23, 1.22	0.134	0.58	0.23, 1.46	0.249
Religiosity *(No)*						
Yes	6.72	3.76, 12.02	**< 0.001**	6.78	3.68, 12.50	**< 0.001**

^*a*^ For overall group differences

There were no significant associations between gender or ethnicity and support or opposition for EAD.

### Qualitative findings

A total of 267 (82%) participants provided answers to the questions about the reasons for their answer to question one, and 225 (69%) described experiences that have influenced their thinking. See Tables 4 and 5 for the themed reasons and experiences respectively, and examples of each divided into year groups.

**Table 4:** Themes illustrating reasons given for answers ‘yes’, ‘no’ or ‘unsure’ to explain responses to questions 1 (section 2)

***Years 2 & 3***	
**Themes for supporting a law change**	**Example**
Relieving suffering	“To stop patient suffering. If a patient is already going to die in 10 days and they are suffering miserably, I think euthanasia has a place by reducing the length of suffering. But I think this is one of the only situations in which it should be used.”
Autonomy	“At the end of the day people deemed “competent” should have no reason to not be able to make a choice about ending their life in a comfortable way if that is what they really desire.”
Enabling a dignified death	“Think people should be able to die with dignity if they want”
Financial reasons	“Thinking about hospital resources it would also save an incredible about of money and free up beds (for people who have a chance to improve their quality of life).”
**Themes for opposing a law change**	**Example**
Potential for misuse	“I do not support a law change due to lack of trust in the system. I see far too many opportunities for health care practitioners (not just doctors) to become ‘trigger happy’ or forceful about euthanasia. I also envision situations wherein patients feel pressured to accept this as an option. There is also quite a lot of room in the system for error and/or abuse of elders and those with disabilities.”
Sanctity of life	“… and I think euthanasia could have a strong follow on effect in the community in regards to how illness and life quality is viewed”
Slippery slope	“Concern over how this could be used and could progress to include mental illness and childhood illness”
Not the role of doctor	“Not the place of the medical profession to carry out this role; does not align with mission; could jeopardise public trust in the profession, with flow-on effects for public health (e.g. not following health advice, avoiding GP and hospitals)”
**Themes for being unsure about a law change**	**Example**
Potential for misuse	“It is very complex. On one hand I don’t have a problem with easing the end for people with progressive conditions. On the other hand I have concerns about how the system will work and be used. My fear is that those with disabilities might be at risk. Or those with mental illness. Or those that see themselves as a burden, rather than actually wanting to end their own suffering, using it as a tool to “end” the suffering of those around them.”
Not the role of doctor	“Furthermore, this changes the perception of doctors. A profession that willingly participates in euthanasia is not one I signed up for.”
General uncertainty	“However, I have lived a fortunate life and have not know anyone to experience a drawn-out, painful death, so I can’t pretend that I know what’s best for people in that position - hence on the fence.”
Relieving suffering	“Because it is a complex issue and people’s lives and connections to others are also complex as could be the influences on the person thought to be voluntarily and competently requesting to do this. On one hand this may well be the compassionate and empathetic thing to provide for a person who is in pain, suffering and terminally ill.”
***Years 4 & 5***	
**Themes for supporting a law change**	**Example**
Relieving suffering	“In some circumstances, the end of life can be very prolonged and difficult and this can worsen suffering for the dying individual and their family.”
Autonomy	“It’s rooted in a fundamental respect of individual’s right to choose for themselves.”
Enabling a dignified death	“It is the single best way to allow patients with debilitating terminal diseases to choose to have a good death.”
Relief for family	“In some circumstances, the end of life can be very prolonged and difficult and this can worsen suffering for the dying individual and their family.”
**Themes for opposing a law change**	**Example**
Potential for misuse	“Having seen abuses of patients within the healthcare system (outside of medical school) and aware as I am of our imperfect and socially unequal society, I am quite nervous about vulnerable people being under pressure either within the healthcare system or by family or personal circumstances to choose assisted dying when it would not have been their choice otherwise.”
Not the role of doctor	“It places doctors in a position where they take life.”
Palliative care as an alternative to EAD	“A comprehensive study regarding the symptoms a person at EOL experience showed that the process of dying itself can be very peaceful with appropriate palliative care input, e.g. pain is less of a concern than the general population would think. We have a robust palliative care system in NZ and I believe we would be demeaning its importance if we legalised euthanasia …” “… Rather than focusing on euthanasia, I think it would be more beneficial in the long-term to focus on a robust palliative care system …”
Personal values	“I personally believe that life is sacred and that no person should be legally allowed to end another person’s life, even at the other person’s request”
**Themes for being unsure about a law change**	**Example**
Undermines palliative care	“I would also want to ensure palliative care wouldn’t be neglected because euthanasia was brought in.”
Not the role of doctor	“Doctors could administer the euthanasia, but I don’t think they should have a role in assessing or deciding if someone should receive euthanasia.”
Potential for misuse	“I predict that assessing competence and the whole process in general may be fraught with danger and difficulties. The person could be persuaded/pressured by family and friends or they may feel pressure to end their life independent of anything the family have actually done.”
Autonomy	“I believe in autonomy - and have met many patients who are terminal and say they want to die. But I think legislating is fraught with difficulty.”

**Table 5:** Themes illustrating the experiences that influenced participants’ views

***Years 2 & 3***	
***Themes generated answering ‘yes’ to supporting EAD***	**Example**
Death of family member or friend	“I have family who have since passed away from complications of dementia and I saw how it negatively impacted my family. I know that my family member who had dementia, would have hated to see herself in that condition.”
Experience in rest home	“I worked in the psychogeriatric ward of a rest home where I saw people who would soil themselves, dribble, become violent etc. I would absolutely HATE to see one of my loved ones or myself in a situation like this.”
Formal medical school teaching (e.g. lectures or tutorials)	“Lectures in ELM2 and ELM3 Palliative care and ethics vertical modules”
Public discussion/personal study	“A lot of debate over in school as we studied a film (which I forgot the name of) which touched on this subject. Have read up on several articles after this.”
***Themes generated answering ‘no’ to supporting EAD***	**Example**
Formal medical school teaching (e.g. lectures or tutorials)	“I previously supported the law, but after discussion with a variety of people and lectures regarding palliative care I’ve come to change my opinion.”
Discussion with family or friends	“My grandparents worrying constantly that they are a burden to their kids and grandkids”
Death/suffering of family member or friend	“A friend committing suicide”
Discussion with doctors	“Spending time with medical physicians and ward nurses who do not support euthanasia for the reasons I have listed above.”
***Themes generated answering ‘unsure’ to supporting EAD***	
Formal medical school teaching (e.g. lectures or tutorials)	“I was set on it being a good thing to legalise until we had our lecture on it in third year where I was forced to practically think about it.”
Public discussion/personal study	“Did a lot of research and debating about the issue in school”
Experience in rest home	“On the other hand, after working in the rest home with many degenerative/debilitating illnesses it should be a choice. I would want the choice.”
Death/Suffering of family member or friend	“I have been with family and non-family deaths. Of adults and children. Hospice and non-hospice. My grandmother’s passing from bowel cancer was managed beautifully by hospice, and with this management euthanasia wasn’t even something that would have been relevant. Children with significant disability and health conditions would be at risk in circumstances where they couldn’t clearly communicate. I just have concerns.”
***Year 4 & 5***	
***Themes generated answering ‘yes’ to supporting EAD***	
Experience in clinical training (e.g. observations of and interactions with patients on ward rounds)	“I have seen palliative patients experience significant suffering prior to their “natural” death e.g. severe pain, intense discomfort, intractable bleeding. I do not believe it is humane to allow suffering in such terminal cases if the patient wishes to end their own life.”
Death/suffering of family member or friend	“My grandfather had a stroke which left him paralyzed. Despite being of sound mind he could no longer move, something which would never improve. The last 6 months of his life were hell, without much joy and he was ready to go. I think he deserved the CHOICE, one last thing he could control if he wished to”
Public discussion/personal study	“Newspaper story about a patient with motor neuron disease who had to resort to starving himself as he no longer wished to live”
Formal medical school teaching (e.g. lectures or tutorials)	“Teaching on degenerative illnesses and palliative care”
***Themes generated answering ‘no’ to supporting EAD***	
Formal medical school teaching (e.g. lectures or tutorials)	“Teaching in medical school have also shaped my opinions around palliative care and given me a lot of confidence in it - I believe it could be better, but I think it is the best way forward.”
Death/suffering of family member or friend	“I have a grandmother who was diagnosed with cancer 5+ years ago that at times has looked very unwell including needing a short stay in hospice care 4 years ago. She is the type of person who does not wish to burden others and I am convinced she would elect for physician assisted dying at the point she was admitted to hospice. She is still alive many years later and has managed to continue living at home with my grandfather who otherwise would be in a residential care facility. This has painted a pretty clear picture as to how vulnerable people’s lives could be ended early under this law change to the detriment of those around them and society in general.”
Discussion with doctors	“Discussions with palliative care specialists”
Public discussion/personal study	“I was pro-euthanasia and the law change prior to the euthanasia debate hosted at the Clinical Leadership Forum earlier this year in Wellington. There were top people in their fields from either side of the argument so it created an ideal environment to form an educated opinion.”
***Themes generated answering ‘unsure’ to supporting EAD***	
Experience in clinical training (e.g. observations of and interactions with patients on ward rounds)	“During my hospice visit in 4th year, we were assigned to visit a palliative patient in their home. I think they intended to show us how palliative care and hospice make a positive impact on patients’ lives, however I ended up visiting a patient who was absolutely depressed and suicidal. They had oesophageal cancer and couldn’t swallow any food which made them miserable. There was nothing the hospice could do to ease their suffering. This was the first time I thought that euthanasia would be a good option.”
Formal medical school teaching (e.g. lectures or tutorials)	“Before I was a medical student I was very for this issue, I wanted it to become legal, because I thought people shouldn’t have their lives drawn out in suffering and have old people just waiting to die. When I became a medical student, the lack of education a lot of patients and family have around what doctors can do and medical ethics is very obvious”
Discussion with family or friends	‘I know that my nan would want to choose assisted dying if she deteriorated to an unsatisfactory condition and then I think assisted dying would be good. But then I think about it as if I was the doctor …’
Public discussion/personal study	“A panel at a medical leaders’ conference that included speakers Ben Gray and David Seymour. The discussions around anti-euthanasia about the emotional impact on doctors, the increase in health inequities and the happiness of patients when palliative care is done right all made me re-evaluate my position. And I haven’t settled on a position since then.”

For both year groups (years 2&3 and 4&5), the authors identified three main themes for the reasons most often cited by those who supported a law change: autonomy, relieving suffering, and enabling a dignified death. Several participants also mentioned the relief it would bring family, that it would provide a safe means for people to end their life who would do so anyway, that palliative care could not resolve all suffering, that current palliative care practice already involves EAD, and financial reasons. The identified themed reasons cited by those who were opposed to a law change were more varied. The most common were that there is a potential for the practice to be misused and that EAD is not compatible with the role of doctors. Several students also mentioned sanctity of life, the potential slippery slope, their own personal values, and that palliative care is an adequate alternative to EAD. Many of those who were unsure cited similar reasons to those who were opposed, but cited these as concerns, while many in this group also expressed sympathy for the reasons given by those who support a law change. There was no notable difference between the ELM and ALM groups in terms of the reasons given.

The themed experiences that participants described as influencing their thinking were largely similar across the ‘yes’, ‘no’ and ‘unsure’ groups, and also year levels. Several in each group mentioned formal medical school teaching activities (such as lectures or tutorials) as influential, and several in ALM referred to experiences in clinical training (e.g. observations of and interactions with patients on ward rounds and in clinics). Discussions with family or friends, discussions with doctors, public lectures and personal study were also cited as influential. The death or suffering of a family member or friend was the experience most frequently mentioned by those who were supportive of a law change in both ELM and ALM. This theme was frequently mentioned for all year groups and all responses except ‘unsure’. A high number of those in ELM (years 2 &3) who supported a law change also mentioned their rest home placement, where second year students work as assistant caregivers in residential care facilities. Those who were opposed to a law change most often identified medical school teaching as having influenced their thinking, and a small number directly stated that their views had changed because of what they have learnt in medical school. Several in all groups demonstrated considerable prior and independent thinking on the issues.

## Discussion

### Quantitative findings

A survey of University of Otago medical students was undertaken to learn their views of EAD, in order to learn how medical education affects medical students’ views of this issue. The results of the survey show that the odds of medical students supporting EAD at 5th year compared to 2nd year is 0.30 (95% CI: 0.15–0.60). The level of support found among 2nd and 3rd year (65 and 63% respectively) students is close to that found in the general public (68%) [3]. However, the level of support among 5th year students (39%) is closer to that of general practitioners in New Zealand (41%) [3]. Identifying with a religion was also found to have a statistically significant correlation with higher levels of opposition and lower levels of support for EAD, which is consistent with other studies [1]. To our knowledge, this is the first study to investigate how New Zealand Medical Students view EAD, and whether their views are affected by medical training.

The level of support among 2nd and 3rd year students is generally higher than that reported in several overseas studies of medical students [11–16], though caution should be taken in drawing precise and direct comparisons as these studies use a variety of questions that are worded differently, and involve students at a range of stages of learning, along with a variety of medical degree structures (e.g. length of degree, undergraduate vs. postgraduate entry).

The similarity between the levels of support for EAD among 2nd and 3rd year participants and the general public supports the link that Stronegger et al 2011 identified between medical students’ views and levels of support in society. However, the difference in support across year levels suggests that medical education may be another significant factor. This suggests that the opposition among practicing doctors is not simply due to social factors at the time they entered medical school, the personality of those who choose to be doctors, or because doctors are naturally more ‘conservative’ on moral issues. If this is true, then we should not expect doctors to become more supportive of EAD in the future, simply because societal attitudes have changed.

Possible alternative explanations for why the 5th year students were less likely to support EAD than the 2nd year students are that simply being older makes a person less likely to support EAD, or that the 5th year group were less supportive when they were 2nd year too, because of social or cultural factors that had since changed. Entry criteria to Otago Medical School were not changed substantially between the times these year groups entered medical school, so the difference is not likely to be due to demographic factors such as socio-economic status. The study synthesising the evidence on New Zealanders’ attitudes to EAD between 1996 and 2017 found that the relationship between age and levels of support was unclear, with some studies reporting lower levels of support among older people and others reporting higher support or no significant difference [3]. In any case, if age was a significant factor, it seems unlikely that it would take effect over 3 years at the stage of life of these participants. The synthesis study also found that support and opposition for EAD in New Zealand was stable between 2002 and 2017 [3]. This suggests that the difference in support is unlikely to be due to sociological differences, and that the most likely explanation is that the students have changed their views during their medical education.

The finding of lower support for EAD among medical students’ further along in their education aligns with findings in three other studies [19–21]. It does not align with the results of one study of students at an English Medical School, which reported that being a final year student was weakly associated with a greater likelihood of agreeing with EAD [16]. However, unlike in this study, a clear majority were opposed to EAD at both the first and final year of training. It seems likely that medical students support and oppose EAD for a range of reasons, and it is possible that medical education only influences some of these reasons. An explanation for why this English study found a different effect could be that the students’ views were generally less changeable.

### Qualitative findings

The reasons participants provided to explain their answers to question one broadly correspond with what is known about the views of the general public in New Zealand [26], and with the reasons typically put forward in the international debate [27]. This suggests that the participants as a group are not substantially different in how they think about EAD from the rest of the population. However, the reasons themselves do not provide a clear indication of why the medical students at 5th year are less likely to support EAD than those at 2nd year. It could be that during medical education a student becomes more aware of reasons to be opposed, or that such reasons become more compelling because of certain clinical experiences they have.

Overall, the experiences that participants identified as influencing their views also do not indicate a clear answer to that question. Though a higher proportion of those who were opposed identified medical school teaching as influential, this was the case for both ELM and ALM students, and some of those who were supportive also identified medical school teaching and experiences in clinical training as having influenced their views. This suggests that it is not any particular part of or event during medical education that changes students’ views, but rather that a variety and perhaps accumulation of experiences can change students’ views in both directions.

### Overall discussion

There are several aspects of medical education that might make medical students less likely to support EAD as they progress. Exposure to end-of-life situations could be a factor. At Otago Medical School students only begin learning in clinical environments with direct patient contact from fourth year. One study attributed decreased support to increased personal experience in end of life care [28]. However, another found that while personal experience in caring for a dying person correlates with less indecision in terms of views, there was no significant correlation between such experience and support or opposition for EAD [29]. Hence, it is perhaps not personal experience with care of the dying that reduces support (e.g. caring for a dying relative), but being professionally educated in, and having a sense of the obligations involved in, providing such care. Specifically, it may be that education in healthcare brings a greater awareness of the treatments and approaches that are available (e.g. how pain can be managed, holistic approaches to suffering), which reduces the sense of a need for EAD [30]. It could also be that medical education increases awareness of the practical challenges or risks involved in applying an EAD policy, either for patients or the practitioners [31]. Studies have reported that practitioners find the processes around EAD stressful and difficult [1].

It is well established that medical students learn about the medical profession from the formal, informal and hidden curriculum [32, 33]. Theoretically, each of these dimensions of the curriculum could influence how participants view EAD. However, it is not clear what part of the formal curriculum would, or could, have this effect. In a Dutch study, student attitudes remained relatively constant before and after a ‘euthanasia and doctor-assisted suicide’ conference, though knowledge improved significantly by the end [34]. When EAD is addressed in ethics classes at Otago Medical School, students are presented with contrasting arguments and encouraged to think independently, and – as noted – medical school teaching was identified as an influential factor by participants representing all three responses (‘yes’, ‘no’ and ‘unsure’).

The concept of the hidden curriculum is that medical education is not just learning about knowledge and skills, but is also a process of socialisation into the medical profession [32, 33]. It is possible that the students are influenced by the doctors they learn from in clinical rotations, and that in their change of views they are conforming to a perceived professional norm. If this is the case, then it might be thought to align with the claim that doctors as a group are more socially or ethically conservative. However, it is also possible that this apparent ‘conservatism’ is grounded in ethical concerns that members of the general public would share if they had a clearer understanding of how doctors perceive this issue. In studies where doctors have been asked why they are opposed to EAD, they often articulate these kinds of reasons, rather than personal values [35, 36]. Furthermore, it is possible that responsiveness to these reasons depends on certain practical knowledge. Drawing on the work of the philosopher Ludwig Wittgenstein, Amesbury has argued that understanding the normative force of reasons requires some familiarity with the practices in which the reasons are used [37]. Perhaps medical students become less likely to support EAD because they gain this familiarity, and so understand the ethical weight of the sorts of reasons put forward by doctors who are opposed. Such practical understanding is often not adequately represented in whatever reasoning a person puts forward to explain their position, and this is perhaps reflected in the absence of any clear difference between the reasons put forward by ELM and ALM participants. If correct, this suggests there is a practical basis to ethical and professional formation, which underpins ethical reasoning.

Further qualitative research may provide deeper insight into which aspects of medical education influence medical students’ views, and why some students appear to change their views during medical education while others do not. Similar research at other medical schools might help to show whether the difference between year groups found in this study is due to local factors or a common feature of medical education.

#### Limitations and strengths

The study was only conducted at one medical school and so the findings may not be generalizable to all medical schools. While there are some studies from other medical schools that have found similar results, comparisons are difficult due to the variables involved. The response rate of this study is low, and nothing is known about the views of those who did not respond. Most other, comparable types of studies have received a similar response rate [20]. The sample is broadly similar to the overall cohort in terms of age, gender, and ethnicity.

Strengths of the study are that it examines the views of medical students across 4 year levels and integrates quantitative and qualitative findings. While the quantitative findings show a difference in views between year groups, the qualitative findings suggest that this difference is not related to a clearly identifiable aspect of medical education, but rather that the participants’ views of this issue are influenced by a range of experiences they have had, and the different ways they have responded to these. A follow up study of the 2nd year cohort once they have completed 5th year may strengthen the findings.

The difference in levels of support between 2nd and 5th year students could be due to a range of factors relating to medical education, including exposure to end of life situations in 4th and 5th years, increased knowledge of the options that are available through palliative care, awareness of the practical challenges created by EAD policy, and socialisation. It is not clear whether the effect is due to local factors or medical education in general.

## Conclusions

Many studies internationally have found that doctors are less likely to support EAD than the general public. It may be thought that this is because of certain personal attributes assumed to be more common among doctors (e.g. being morally conservative), but it could also be because doctors perceive reasons to not support EAD that others would share if they had the same practical knowledge. This survey of Otago Medical School students’ views of EAD found that students at the end of 5th year were less likely to support EAD than those at the end of 2nd year. These findings suggest that the difference in support between doctors and the general public is not simply attributable to personal factors. These conclusions should be considered when interpreting public opinion surveys on this issue. The findings also suggest a practical basis to ethical and professional formation, which – if correct – should inform teaching about professionalism and medical ethics. Further research on medical students’ views of EAD may reveal more about how those views are formed during medical education, and which aspects of their learning make them less likely to be supportive.

## Supplementary Information

**Additional file 1: Appendix. Tables 6 & 7 and Questionnaire** a Demographics of survey respondents and cohort, and Questionnaire.

## Data Availability

The datasets generated and analysed during the current study are not publicly available due to privacy reasons, but are available from the corresponding author on reasonable request.

## Abbreviations

EAD: Euthanasia/assisted dying
HSFY: Health Sciences First Year (a year of study through which most students enter Otago Medical School)
ELM: Early Learning in Medicine (2 years of pre-clinical learning at Otago Medical School)
ALM: Advanced Learning in Medicine (three clinical years of learning at Otago Medical School)

## Footnotes

1 Disagreement over which terms should be used to describe particular actions is part of the wider debate of this issue. ‘Euthanasia’ is the more traditional terms, while proponents often prefer the term ‘assisted dying’, or variations of this. Throughout this discussion we use the abbreviation EAD to refer to the range of actions that might be considered within the debate, including administering a drug with the intention of ending a person’s life, and supplying a person with the means to end their own life. The survey question that we used specified a particular form of action (see below).
