# Supplementary material for: New Zealand medical students’ views of euthanasia/assisted dying across different year levels
Source: BMC Med Educ. 2021 Feb 23;21:125. doi: 10.1186/s12909-021-02558-2 (PMC7901115; doi:10.1186/s12909-021-02558-2)
Supplement: Supplementary file 1 — Additional file 1: Appendix. Tables 6 & 7 and Questionnaire a Demographics of survey respondents and cohort, and Questionnaire. [file 12909_2021_2558_MOESM1_ESM.docx]

Title: **New Zealand medical students’ views of euthanasia/assisted dying across different year levels**

Authors: **Luke Nie**^1^, **Kelby Smith-Han**^1^, **Ella Iosua**^1^, **Simon Walker**^1✉^

^1^ University of Otago, Dunedin, New Zealand

*Correspondence*

Simon Walker, University of Otago, 362 Leith St, North Dunedin, Dunedin 9016, New Zealand.

Email: [simon.walker@otago.ac.nz](mailto:simon.walker@otago.ac.nz)

*Email addresses of co-authors:*

Luke Nie: nielu538@student.otago.ac.nz

Kelby Smith-Han: kelby.smith-han@otago.ac.nz

Ella Iosua: ella.iosua@otago.ac.nz

**Appendix**

**Table 6 – demographics of survey respondents**

|  |  | 2^nd^ year  (N=105) | 3^rd^ year  (N=91) | 4^th^ year  (N=66) | 5^th^ year  (N=64) |
| --- | --- | --- | --- | --- | --- |
| Age | 18-24 | 94 (89.5) | 84 (92.3) | 52 (78.8) | 48 (73.9) |
|  | 25-29 | 3 (2.9) | 6 (6.6) | 6 (9.1) | 11 (16.9) |
|  | 30-39 | 7 (6.7) | 1 (1.1) | 6 (9.1) | 5 (7.8) |
|  | 40+ | 1 (1.0) | 0 (0.0) | 2 (3.0) | 0 (0.0) |
| Gender | Female | 63 (60) | 63 (69.2) | 43 (65.2) | 30 (46.9) |
|  | Male | 41 (39.1) | 27 (29.7) | 22 (33.3) | 33 (51.6) |
|  | Gender diverse | 1 (1.0) | 1 (1.1) | 1 (1.5) | 0 (0.0) |
|  | Prefer not to say | 0 (0.0) | 0 (0.0) | 0 (0.0) | 1 (1.6) |
| Ethnicity | NZ European | 73 (69.5) | 65 (72.2) | 47 (72.3) | 47 (74.6) |
|  | Māori | 17 (16.2) | 17 (18.9) | 8 (12.3) | 10 (15.9) |
|  | Samoan | 4 (3.8) | 1 (1.1) | 2 (3.1) | 2 (3.2) |
|  | Cook Island Māori | 0 (0.0) | 2 (2.2) | 0 (0.0) | 0 (0.0) |
|  | Tongan | 0 (0.0) | 0 (0.0) | 0 (0.0) | 2 (3.2) |
|  | Niuean | 0 (0.0) | 0 (0.0) | 0 (0.0) | 1 (1.6) |
|  | Chinese | 7 (6.7) | 11 (12.2) | 4 (6.2) | 4 (6.3) |
|  | Indian | 3 (2.9) | 3 (3.3) | 2 (3.1) | 2 (3.2) |
|  | Other | 20 (19.0) | 12 (13.3) | 13 (20.0) | 7 (11.1) |
| Ethnicity | Māori | 17 (16.2) | 17 (18.9) | 8 (12.3) | 10 (15.9) |
|  | Non- Māori | 88 (83.8) | 73 (81.1) | 57 (87.7) | 53 (84.1) |
| Religion | No religion | 73 (69.5) | 65 (72.2) | 42 (64.6) | 42 (65.6) |
|  | Christian | 26 (24.8) | 22 (24.4) | 18 (27.7) | 17 (26.6) |
|  | Buddhist | 1 (1.0) | 1 (1.1) | 1 (1.5) | 0 (0.0) |
|  | Hindu | 3 (2.9) | 0 (0.0) | 1 (1.5) | 1 (1.6) |
|  | Muslim | 0 (0.0) | 0 (0.0) | 1 (1.5) | 2 (3.1) |
|  | Jewish | 0 (0.0) | 0 (0.0) | 0 (0.0) | 0 (0.0) |
|  | Other religion | 1 (1.0) | 2 (2.2) | 1 (1.5) | 0 (0.0) |
|  | I object to answering | 1 (1.0) | 0 (0.0) | 1 (1.5) | 2 (3.1) |
| Religious | Yes | 31 (29.8) | 25 (27.8) | 22 (34.4) | 20 (32.3) |
|  | No | 73 (70.2) | 65 (72.2) | 42 (65.6) | 42 (67.7) |

**Table 7 – demographics of Otago Medical School cohort**

|  |  | 2^nd^ year  No. (%) | 3^rd^ year | 4^th^ year | 5^th^ year |
| --- | --- | --- | --- | --- | --- |
| Age | 18-24 | 267 (90.2) | 259 (89.3) | 239 (80.7) | 202 (74.8) |
|  | 25-29 | 17 (5.7) | 21 (7.2) | 34 (11.5) | 45 (16.7) |
|  | 30-39 | 12 (4.1) | 9 (3.1) | 18 (6.1) | 22 (8.1) |
|  | 40+ | 0 (0) | 1 (0.3) | 5 (1.7) | 1 (0.4) |
|  | | | | | |
| Gender | Female | 162 (54.7) | 178 (61.4) | 177 (59.8) | 145 (53.7) |
|  | Male | 134 (45.3) | 112 (38.6) | 119 (40.2) | 125 (46.3) |
|  | Gender diverse | 0 (0) | 0 (0) | 0 (0) | 0 (0) |
|  | Prefer not to say | 0 (0) | 0 (0) | 0 (0) | 0 (0) |
|  | | | | | |
| Ethnicity | NZ European | 195 (65.9) | 191 (65.9) | 187 (64.1) | 185 (68.5) |
|  | Māori | 56 (18.9) | 58 (20.0) | 50 (16.9) | 41 (15.2) |
|  | Samoan | 18 (6.1) | 6 (2.1) | 10 (3.4) | 3 (1.1) |
|  | Cook Island Māori | 1 (0.3) | 5 (1.7) | 4 (1.4) | 4 (1.5) |
|  | Tongan | 3 (1.0) | 3 (1.0) | 3 (1.0) | 1 (0.4) |
|  | Niuean | 4 (1.4) | 1 (0.3) | 0 (0.0) | 0 (0.0) |
|  | Chinese | 27 (9.1) | 28 (9.7) | 29 (10.0) | 26 (9.6) |
|  | Indian | 11 (3.7) | 11 (3.8) | 18 (6.2) | 11 (4.1) |
|  | Other | 73 (24.7) | 80 (27.6) | 82 (27.9) | 72 (26.7) |

**Questionnaire (administered via Qualtrix)**

Thank you for participating in this survey. The answers you provide will remain anonymous.

*Section 1*

1. What year of medicine did you complete in 2018?
2. 2^nd^ Year
3. 3^rd^ Year
4. 4^th^ Year
5. 5^th^ Year
6. What is your age?
7. 18-24
8. 25-29
9. 30-39
10. 40+
11. What is your gender?
12. Male
13. Female
14. Gender diverse
15. Prefer not to say
16. Which ethnic group do you belong to?
17. New Zealand European
18. Māori – Which iwi do you belong to? [open text response]
19. Samoan
20. Cook Island Māori
21. Tongan
22. Niuean
23. Chinese
24. Indian
25. Other such as Dutch, Japanese, Tokelauan. Please state: [open text response]
26. What is your religion?
27. No religion
28. Christian - which denomination? (Anglican, Catholic, Presbyterian, Ringatū, Non-denominational etc.) [open text response]
29. Buddhist
30. Hindu
31. Muslim
32. Jewish
33. Other religion: [open text response]
34. I object to answering this question
35. How often do you attend church/synagogue/mosque/or engage in other spiritual activities?
36. Never
37. Daily
38. Weekly
39. Monthly
40. Annually
41. Do you have experience working in a healthcare profession?
42. Yes – which profession? [open text response]
43. No

*Section 2*

1. The End of Life Choice Bill is presently being considered by a parliamentary select committee. It would make it lawful, in certain circumstances, for doctors to provide or administer a medicine to a competent person, at their request, that will bring about their death.

**Do you think the law in New Zealand should be changed to allow doctors, under certain circumstances, to provide or administer a medicine to a person, at their voluntary and competent request, that will bring about their death?**

1. Yes
   1. Could you please state your main reasons for supporting a law change? [open text response]
   2. Under what circumstances do you think this should be legal? Please list: [open text response]
2. No
   1. Could you please state your main reasons for not supporting a law change? [open text response]
3. Unsure
   1. Why are you unsure about this issue? [open text response]
   2. What questions do you have about this issue, which, if answered, might clarify your position? [open text response]

1. Could you please briefly describe any particular experiences that have influenced your thinking on this issue? (This may include teaching and learning experiences at medical school, or personal experiences) [open text response]
2. In order to explore this issue further, we would like to interview medical students about their views. Would you be willing to be interviewed?
   1. Yes
      1. Please insert your email address. [open text response]

(Your privacy will be respected. Data will be anonymized before presentation to the rest of the research team. Your responses will remain anonymous in any reporting or publication of this study.)

- 1. No

1. To be in the draw to win one of two $50 supermarket vouchers, please enter your student ID number: [open text response]
